# Clinical clerkship students’ preferences and satisfaction regarding online lectures during the COVID-19 pandemic

**DOI:** 10.1186/s12909-021-03096-7

**Published:** 2022-01-18

**Authors:** Shusuke Yagi, Daiju Fukuda, Takayuki Ise, Koji Yamaguchi, Kenya Kusunose, Muneyuki Kadota, Yutaka Kawabata, Tomomi Matsuura, Tomohiro Soga, Hirotsugu Yamada, Takeshi Soeki, Tetsuzo Wakatsuki, Shinji Kawahito, Masataka Sata

**Affiliations:** 1 Department of Cardiovascular Medicine, Tokushima University Graduate School of Biomedical Sciences, 3-18-15 Kuramoto-cho, Tokushima, 770-8503 Japan; 2 Department of Community Medicine and Human Resource Development, Tokushima University Graduate School of Biomedical Sciences, Tokushima, Japan; 3 Department of Community Medicine for Cardiology, Tokushima University Graduate School of Biomedical Sciences, Tokushima, Japan

**Keywords:** Clinical clerkship, COVID-19, Online lectures

## Abstract

**Background:**

The COVID-19 pandemic has caused an unprecedented disruption in medical education. Students and lecturers had to adapt to online education. The current study aimed to investigate the level of satisfaction and future preference for online lectures among clinical clerkship students and elucidated the factors that affect these outcomes.

**Methods:**

We selected a sample of 114 medical students undergoing clinical clerkship during the COVID-19 pandemic. We conducted onsite lectures before the pandemic and online lectures after the outbreak. A survey was conducted, and the sample included students and 17 lecturers. The average scores of total satisfaction and future preference related to online lectures were computed.

**Results:**

Students’ scores on total satisfaction with online lectures and their future preference were higher than those for onsite lectures. Scores on the ease of debating dimension were low and those on accessibility of lectures in online lectures were higher than those in onsite lectures. There was no difference between the two groups in the scores on the comprehensibility and ease of asking questions dimensions. Results of the multiple regression analysis revealed that accessibility determined total satisfaction, and future preference was determined by comprehensibility as well as accessibility. Contrary to students’ future preferences, lecturers favored onsite lectures to online ones.

**Conclusion:**

Online lectures are an acceptable mode of teaching during the COVID-19 pandemic for students undergoing clinical clerkship. Online lectures are expected to become more pervasive to avoid the spread of COVID-19.

## Background

The COVID-19 pandemic has caused an unprecedented disruption in medical education. Students attended lectures on-site before the pandemic [1–3]. However, after the outbreak, the authorities restricted their entry in the university hospital area and recommended online classes [4]. The disruption of medical education on-site caused by the COVID-19 pandemic resulted in mental health disorders, such as anxiety, depression, and burnout syndrome, not only among health care workers but also among medical students. This is because it was difficult to share medical information with colleagues due to the risk associated with COVID-19 infection [5, 6]. The disruption of medical education might have also made medical students feel their delay of progress in medical training or studying. Thus, an alternative way of learning was needed for medical students. Hence, medical students and lecturers in Japanese medical universities had to adapt to online lectures. Online lectures are a safe alternative to onsite classes and have been known to be effective in knowledge acquisition [7]. Some studies have reported that online lectures have been well-received by undergraduate medical students and have been perceived to be as effective, useful, and enjoyable as traditional teaching [8, 9]. Thus, online lectures can improve learning outcomes [10].

However, it remains debatable whether online lectures are effective and preferable didactically over onsite lectures for clinical clerkship students [11, 12]. Additionally, lecturers’ preferences regarding the mode of lectures have not yet been explored.

This study aimed to explore the level of satisfaction and future preferences of students undergoing clinical clerkship with regard to online lectures, and also analyzed the determining factors.

## Materials and methods

### Population

**Fig. 1:**
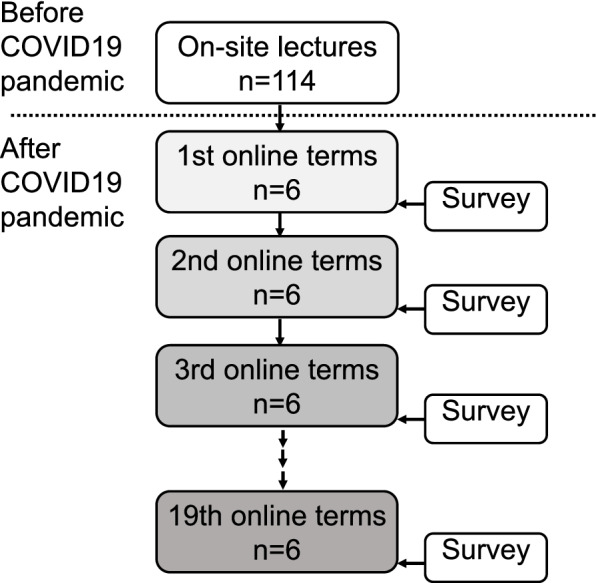
Illustration of the study protocol

Out of all fifth-grade medical students undergoing clinical clerkship at Tokushima University of Medicine from April 2020, 114 students finally participated in the study through convenience sampling (Fig. 1). All participants had attended only onsite lectures before the pandemic and switched to hybrid lectures comprising online and onsite classes after the outbreak. Each group consisted of six students who studied cardiovascular medicine for 2 weeks on a rotational basis. The first group that visited the cardiovascular department and attended our lectures were designated as the first online term.

### Procedures

We conducted online lectures on topics related to cardiovascular medicine including physical examination, ischemic heart disease, heart failure, arrhythmia, intravascular imaging, and echography during the term.

The lectures were delivered in eight sessions, and the duration of each session was between 30 to 60 min. Our university signed a license contract with Microsoft Office 365. Thus, the online lectures were delivered using Microsoft Teams, as per the university guidelines.

The lecturer and the students used their personal computers that were off-line to the intranet of medical records. It was mandatory for students to turn on their videos to confirm their attendance during the lecture, and the lecturer could also observe all students during presentations.

### Measures

We defined total satisfaction and future preference as the outcomes. We conducted online face-to-face survey using Microsoft Teams for students and computed the average scores for satisfaction and preference. Other questions in the survey studied factors including the degree of comprehensibility, ease in asking questions, ease in debating, and accessibility of online and onsite lectures (Table 1).

**Table 1:** Questions for medical students and lecturers

Questions for medical students	
**Question 1**	
How would you rate online lectures on a scale of 1 to 10 on the dimensions total satisfaction and future preference, if the score of on-site lectures was 5 points?	
**Question 2**	
How would you rate online lectures on a scale of 1 to 10 on the degree of comprehensibility of online lectures, if the score of on-site lectures was 5 points?	
**Question 3**	
How would you rate online lectures on a scale of 1 to 10 on the degree of ease of asking questions to lectures, if the score of on-site lectures was 5 points?	
**Question 4**	
How would you rate online lectures on a scale of 1 to 10 on the degree of ease of debating with students and lectures, if the score of on-site lectures was 5 points?	
**Question 5**	
How would you rate online lectures on a scale of 1 to 10 on the degree of accessibility of online lectures, if the score of on-site lectures was 5 points?	
**Questions for lecturers**	
How would you rate online lectures on a scale of 1 to 10 on the total satisfaction and future preference, if the score of on-site lectures was 5 points?	

The score of onsite lectures was fixed at 5 points. Given this, the students were asked to rate the online lectures from 1 to 10. The factors mentioned above were compared for both modes of teaching.

Additionally, 17 lecturers who had conducted the online lectures were asked to share their future preferences with regard to the mode of teaching. On the basis of their responses, the degree of satisfaction and preference were calculated.

All procedures were conducted in accordance with the guidelines released by the Curriculum Coordination Committee of the Faculty of Medicine, Tokushima University. The study protocol was approved by the Ethics Committee of Tokushima University Hospital.

### Statistical analyses

We calculated the average (mean) scores obtained on the questions as ordinal variables. Values were expressed as means ± standard deviations. Differences between online and onsite lectures were analyzed using the paired t-test. The degrees of association among independent variables for total satisfaction and future preference were assessed through multiple regression analysis. All statistical analyses were performed using the JMP (version 11; SAS Institute, Cary, NC) software. The level of statistical significance was set as *p* < 0.05.

## Results

A total of 114 students (79 males and 35 females) were pursuing clinical clerkship, out of which, eight students faced difficulty in attending the online lectures because of device failure and unstable signal; nevertheless, eventually, all students managed to attend all the lectures. Those who faced issues either replaced the device or changed the location from where they were attending the lectures.

**Fig. 2:**
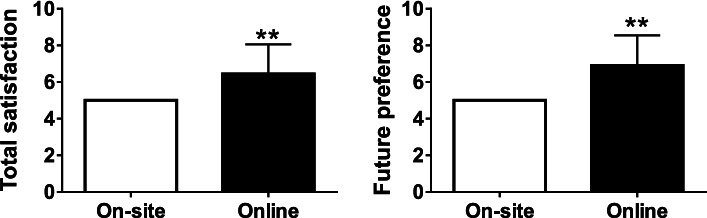
Total satisfaction and future preference of the students as a primary outcome. Points for onsite lectures were fixed at 5 as a reference. ** *P* < 0.01

**Fig. 3:**
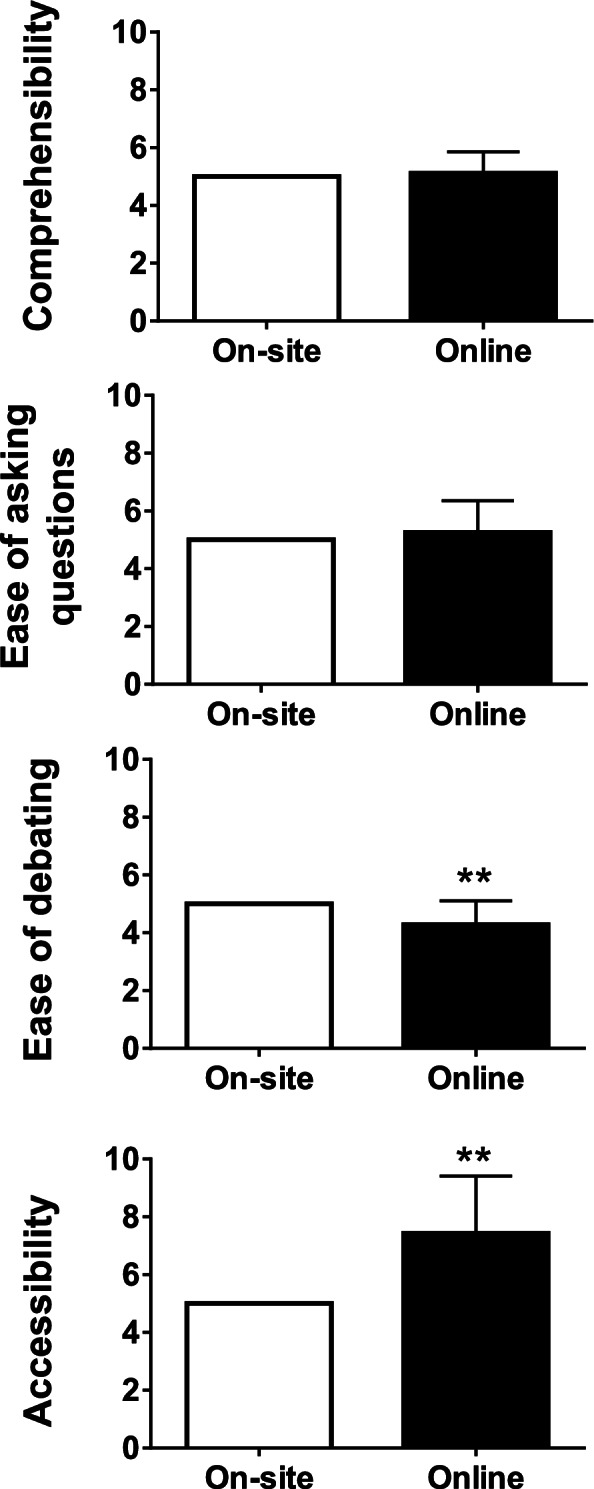
Comprehensibility, ease of asking questions, ease of debating, and accessibility as secondary outcomes. Points for onsite lectures were fixed at 5 as a reference. ** *P* < 0.01

Students’ scores for total satisfaction and future preference for online lectures were significantly higher than those for onsite lectures (Fig. 2). However, the scores for comprehensibility and ease of asking questions for the two modes did not differ significantly. Furthermore, students’ scores for ease of debating in online lectures were significantly low (*P* < 0.01), whereas scores for accessibility of lectures were significantly high, compared to onsite scores (*P* < 0.01) (Fig. 3).

**Fig. 4:**
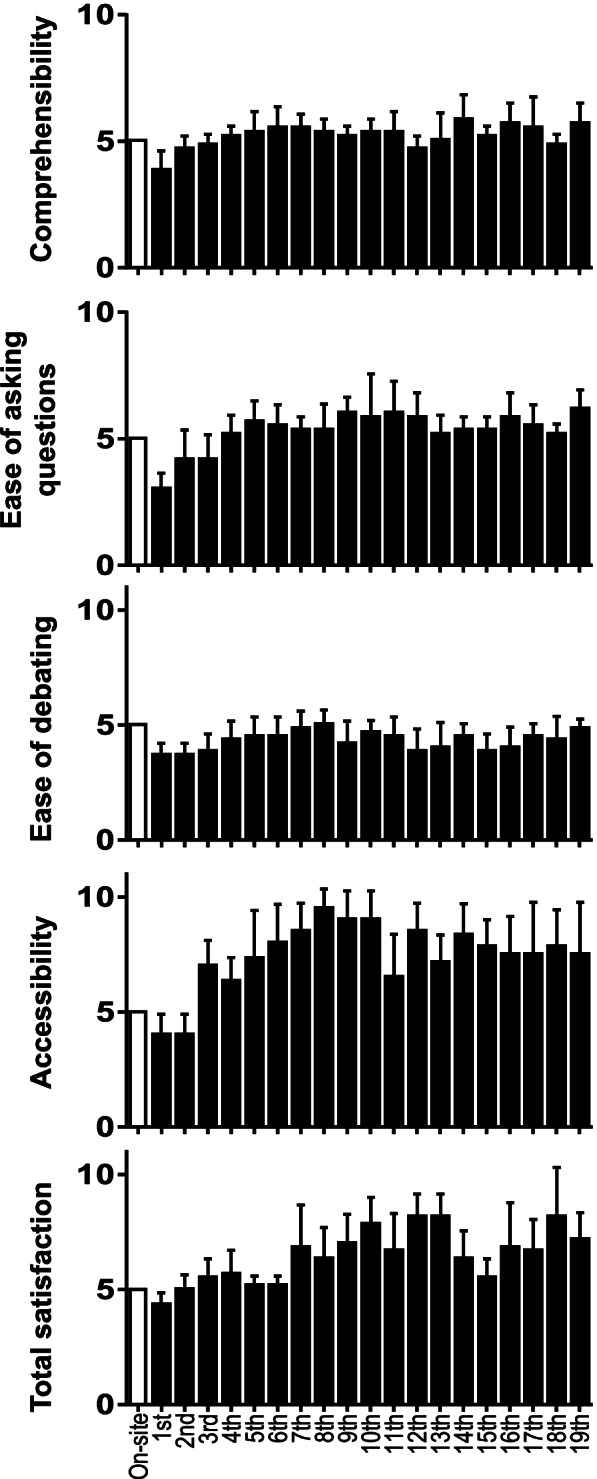
Comprehensibility, ease of asking question, ease of debating, and accessibility over the course of time. Points for onsite lectures were fixed at 5 as a reference

**Fig. 5:**
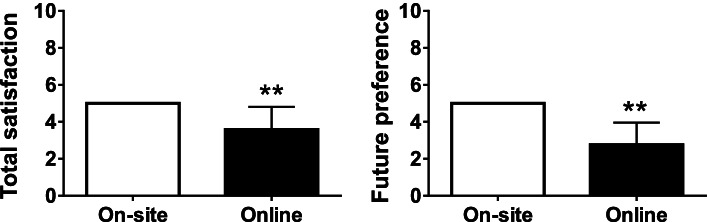
Future preference of the lecturers. Points for onsite lectures were fixed at 5 as a reference. ** *P* < 0.01

The scores for comprehensibility and ease of debating in online lectures in the early-term group tended to be lower than those in the late-term group. The scores for ease of asking questions, especially accessibility to the lectures and total satisfaction tended to improve in the late-term group as compared to the early-term group, indicating that familiarity with online lectures caused an improvement in these factors (Fig. 4). In contrast, lecturers preferred onsite lectures to online ones (Fig. 5).

Results of the multiple regression analysis for the students revealed that accessibility determined total satisfaction (*p* < 0.05; Table 2), and both comprehensibility (*p* < 0.05) and accessibility (*p* < 0.001) determined future preferences (Table 3).

**Table 2:** Multiple regression analysis for determinants of total satisfaction

Variables	Coefficient	95% Confidence Interval	*P*-value
Comprehensibility	0.1	-0.3 to 0.5	0.69
Ease of asking questions	0.3	-0.1 o 0.6	0.11
Ease of debating	0.1	−-0.3 to 0.5	0.56
Accessibility	0.2	0.1 to 0.4	0.03

**Table 3:** Multiple regression analysis for determinants of future preference

Variables	Coefficient	95% Confidence Interval	*P*-value
Comprehensibility	0.5	0.1 to 0.9	0.02
Ease of asking questions	0.1	-0.2 to 0.4	0.69
Ease of debating	0.1	-0.2 to 0.4	0.35
Accessibility	0.3	0.1 to 0.5	<0.001

## Discussion

The results revealed that online lectures were well-received by the students undergoing clinical clerkship, and they preferred them to onsite lectures mainly because of ease of access—contrary to the lecturers’ responses.

During the early term, both lecturers and students were not familiar with the new telecommunication method, especially use of the Teams application, which resulted in low scores for the total satisfaction and future preference outcomes. However, gradually, familiarity with the online mode increased, which was indicated in the increased scores for the outcomes. Thus, familiarity with the telecommunication method could solve minor problems, such as difficulty in using Teams and an unstable online signal, which was reflected in the results of the study, that indicated that there were only minor problems pertaining to the method of conducting lectures amid the COVID-19 pandemic [13].

There was no significant difference in the understanding of the lectures delivered through online and onsite modes, which is the most important factor in teaching [14]. The quality of online instruction did not differ significantly from that delivered onsite when the lecturer was visible on the screen while they presented the slides [15].

In the early term, students hesitated in questioning the lecturers. However, they became less reluctant in asking questions or using the chat function during the later term. This implies that the chat option may be helpful in overcoming the hesitation in asking questions [16].

The ease of debating with the lecturer or other students in the online mode was less than that in onsite lectures. Some students claimed that it was difficult to understand who was being addressed, even though they could see the students and the lecturer via multi-screen feature. Difficulty in assessing the situation might be one of the drawbacks of online teaching. Accessibility would improve in an online format as students become more accustomed to online lectures. Further qualitative studies to understand the student experience may reveal improved findings in such a small group.

The most striking difference between the two modes was in terms of accessibility, which contributed to the total satisfaction and future preference outcomes. Students could easily attend online lectures from their homes. Given the fact that attendance is important for success in medical education [17], accessibility should also be considered as an important factor for success in the telecommunication era.

The preference and satisfaction of lecturers and students clearly depend on the circumstances in which they provide and attend lectures, respectively [11]. Surrounding noise is one of the problems that can lead to disruption of an online lecture. Use of headsets that have a noise cancelation function and microphones might solve the issue of having limited space within a room. Eye fatigue due to constant exposure to a screen for a long time is another problem associated with online teaching. To overcome this challenge, lecturers should pay attention to the number of lectures organized per day and their duration. Hybrid classes (i.e., a combination of onsite and online modes) with optimal number of lectures in both modes might improve students’ academic achievement [18].

In contrast to the students’ future preference, the lecturers preferred onsite classes; this may be because they themselves had attended only onsite lectures as medical students. The generation gap between students and lecturers should be bridged though proper training for lecturers in the telecommunication method [19]. The current teaching methodology might benefit from a paradigm shift in didactics.

This study also had some limitations. First, the number of participants was relatively small, and we could not determine the target sample size because the population at a single site was limited. Second, discussing the online questionnaires with the lecturer brought in some bias in the answers. Third, the limited questions could not gauge the exact quality of the lectures. Fourth, we could not evaluate the onsite and online lectures by using same scales because the timings of onsite and online lectures were different, and they were held during different periods. Additionally, time is also an important determinant, and the quality of lectures improved with time as the students and lecturers became more accustomed to them. Fifth, the quality of lectures was not standardized because the study was observational in nature. Lastly, we did not have a randomized control group. Randomized studies of online and onsite lecture groups are further needed to clarify the efficacy of online lectures.

Online lectures are expected to become more pervasive to avoid the spread of COVID-19. Through this study, we can conclude that the online mode is acceptable for teaching medical students undergoing clinical clerkship.

## Data Availability

The datasets generated during and analyzed during the current study are not publicly available due to their containing information that could compromise the privacy of research participants but are available from the corresponding author on reasonable request.
